# Copper Resistance Mechanism and Copper Response Genes in *Corynebacterium crenatum*

**DOI:** 10.3390/microorganisms12050951

**Published:** 2024-05-08

**Authors:** Mingzhu Huang, Wenxin Liu, Chunyan Qin, Yang Xu, Xu Zhou, Qunwei Wen, Wenbin Ma, Yanzi Huang, Xuelan Chen

**Affiliations:** 1 National R&D Center of Freshwater Fish Processing, Jiangxi Normal University, Nanchang 330022, China; huangmingzhu12@163.com (M.H.); 18065616241@163.com (W.L.); huangyanzi618@163.com (Y.H.); 2 School of Life Science, Jiangxi Normal University, Nanchang 330022, China; 15090135694@163.com (C.Q.); xuyang_2024sun@163.com (Y.X.); 18770194298@163.com (X.Z.); w33570488562004@163.com (Q.W.); mawenbin0104@163.com (W.M.)

**Keywords:** copper stress, bioremediation, *Corynebacterium crenatum*, transcriptome, surface display

## Abstract

Heavy metal resistance mechanisms and heavy metal response genes are crucial for microbial utilization in heavy metal remediation. Here, *Corynebacterium crenatum* was proven to possess good tolerance in resistance to copper. Then, the transcriptomic responses to copper stress were investigated, and the vital pathways and genes involved in copper resistance of *C. crenatum* were determined. Based on transcriptome analysis results, a total of nine significantly upregulated DEGs related to metal ion transport were selected for further study. Among them, GY20_RS0100790 and GY20_RS0110535 belong to transcription factors, and GY20_RS0110270, GY20_RS0100790, and GY20_RS0110545 belong to copper-binding peptides. The two transcription factors were studied for the function of regulatory gene expression. The three copper-binding peptides were displayed on the *C. crenatum* surface for a copper adsorption test. Furthermore, the nine related metal ion transport genes were deleted to investigate the effect on growth in copper stress. This investigation provided the basis for utilizing *C. crenatum* in copper bioremediation.

## 1. Introduction

While copper plays essential roles in the catalysis of metabolic processes as cofactors for all living cells, chronic exposure to excess copper eventually leads to sulfhydryl depletion or generates reactive oxygen species and thereby becomes toxic for organisms [1]. Industrial production has led to increases in the release of large quantities of copper-containing industrial wastewater into the environment, which are considerably harmful to ecosystems and human health [2]. Thus, efficient copper remediation is highly important. Normally, copper removal in industry has primarily relied on physical and chemical methods. Although the conventional physicochemical methods are effective and accurate, most of them require complex instruments and high cost [3]. Therefore, microbiological treatment has become an attractive alternative because of its low cost and eco-friendliness [4].

Heavy metal resistance mechanisms and heavy metal response genes are crucial to microbial utilization for heavy metal remediation. The majority of heavy metal resistance mechanisms are inducible by the cognate metal, and the resistance genes are controlled by the transcriptional regulatory system, which enables the expression of resistance genes only at times of metal stress [5]. The transcriptome investigation is an effective tool for understanding how the changed expression of genes contributes to complex biological processes from a global view during metal stress [6]. Transcriptome analysis can provide further insights into the molecular details that regulate the physiological processes of the cell. Nonetheless, up to now, only a few transcriptome research explored the copper resistance mechanisms of bacteria, such as *Cupriavidus gilardii* CR3, *Escherichia coli*, and *Pseudomonas aeruginosa*. The envelope stress, superoxide stress response, and iron homeostasis increased transcription in response to elevated copper concentrations in *E. coli. P. aeruginosa* quickly responded to copper stress with metabolism reduction. The transcription of *P. aeruginosa* also revealed a Cu^+^-binding periplasmic chaperone, three Cu^+^ efflux systems, and two cytoplasmic CopZ proteins [7,8].

In *Enterococcus hirae*, the *cop* system plays an important role in copper homeostasis. In copper stress, CopZ (copper chaperone) donates Cu^+^ to CopY (the repressor), resulting in a derepression of the cop operon and subsequently in copper export by CopB (copper ATPases). In *E. coli*, copper homeostasis is achieved through the Cus two-component system. In the presence of elevated copper concentrations, CueR activates the transcription of *copA* (a copper ATPases gene) and *cueO* (multi-copper oxidase gene), encoding a P-type ATPase and an oxygen-dependent multicopper oxidase, respectively. In *Mycobacterium tuberculosis*, CsoR (the repressor) loses its DNA-binding affinity, resulting in the derepression of the cso operon and the export of copper via CtpV (copper exporter). These systems play crucial roles in copper efflux and effectively protect cells against copper cellular toxicity. The understanding of the bacterial copper-resistance system is rather fragmentary, and the bacterial copper-resistance mechanisms are relatively species-dependent [8]. Thus, investigating the copper-resistance mechanisms in more bacterial species through transcriptome is essential.

Although several microorganisms isolated from natural sources have been developed for environmental heavy metal remediation, the efficiency of natural microorganisms normally could not meet industrial requirements. Thus, scientists have been improving the technologies of microbial bioremediation to meet industrial demands. Genetically modified bacteria are unquestionably more efficient in the processes of heavy metal remediation [9]. Genetic modification requires a clear genetic background and mature genome editing technology. In addition, the success of utilizing genetically engineered strains depends on the survival of these strains under ecological stress conditions for heavy metal remediation. Some foreign genes have been inserted into the genome of these microorganisms possessing the ability to remove heavy metals in the ecosystem. A few examples of microorganisms that have been genetically engineered include *E. coli*, *Pseudomonas putida*, *Mycobacterium marinum*, and *Bacillus idriensis* [10]. *Corynebacterium crenatum* is a typical industrial Gram-positive bacterium from soil [11]. The genome of *C. crenatum* was sequenced, and it possesses mature genome editing technology [12]. In addition, *C. crenatum* was proven to possess good tolerance in resistance to copper. Therefore, it is very suitable as a genetically engineered bacterium for copper remediation.

In this study, the growth of *C. crenatum* during copper stress was first evaluated. Then, the transcriptomic responses to copper stress were explored, and the vital pathways and genes involved in copper resistance in *C. crenatum* were determined. Some genes were selected to further analyze the differential expression between with or without copper treatment to verify the reliability of the transcriptome analysis results. On the basis of these results, nine gene-deletion mutants were constructed to investigate the effect on the growth in copper stress, and two transcription factors were studied for the function of regulatory gene expression. Furthermore, three copper-binding peptides were displayed on the *C. crenatum* surface for a copper adsorption test. This investigation provided the basis for utilizing *C. crenatum* for enhanced bioremediation of copper.

## 2. Materials and Methods

### 2.1. Strains, Cultivation Conditions, and Plasmids

All plasmids and strains in this work are listed in Table S1. *E. coli* DH5α and *C. crenatum* were cultured at 37 °C and 30 °C, respectively, in Luria–Bertani (LB) medium (Oxoid, Hants, UK). According to the requirement of antibiotics, kanamycin and chloramphenicol (Solarbio, Beijing, China) were applied at doses of 20 and 12.5 mg/L for *E. coli* and *C. crenatum*, respectively. The strains harboring pXMJ19 were cultured with an additional 0.2 mM of isopropyl-β-D-thiogalactoside (IPTG) to induce protein expression. Brain–heart infusion medium (Solarbio, Beijing, China) was applied after electroporation to transform plasmids into *C. crenatum*. pK18mob*sacB* (a deletion vector contained the *sacB*-based suicide gene) was removed using sucrose medium [13].

### 2.2. RNA Preparation and Transcriptome Sequencing

The cells were cultured either with or without 0.5 mM CuSO_4_ as a test group and control group at OD_600nm_ = 1.5 (the exponential phase) for 0.5 h. The total RNA was isolated using the TRIzol reagent (Invitrogen, Carlsbad, CA, USA) in accordance with the instructions, and DNA was removed using DNase (Takara, Beijing, China) for 1 h at 37 °C. After that, the RNA was quantified using ND-2000 (Thermo Fisher, Waltham, MA, USA) by measuring the ratios of A230/260 nm and A260/280 nm, and RNA integrity was verified using agarose gel electrophoresis. The transcriptome library was constructed using the TruSeq RNA sample preparation kit (Illumina, San Diego, CA, USA). Ribosomal RNA was removed using the Ribo-Zero Magnetic kit (Epicenter, Madison, WI, USA). All mRNAs were broken into short fragments, and then cDNA was synthesized using the SuperScript double-stranded cDNA synthesis kit (Invitrogen, Carlsbad, CA, USA). Short fragments were purified with a QiaQuick PCR extraction kit and resolved with EB buffer for end repair and to add poly(A) tails. Then, the short fragments were ligated to sequencing adapters. For amplification with PCR, we selected suitable fragments as templates after agarose gel electrophoresis. The RNA-seq transcriptome library was constructed using a library construction kit (Illumina, San Diego, CA, USA) in accordance with the protocol. Then, it was sequenced in Illumina Novaseq (Illumina, San Diego, CA, USA). The transcriptome library was deposited into the National Center for Biotechnology Information (NCBI, https://www.ncbi.nlm.nih.gov, access number PRJNA1017422).

### 2.3. Differentially Expressed Gene (DEG) Analysis and Functional Annotation

The transcript expression levels of RNA were deduced according to the fragments per kilobase of transcript per million mapped reads (FPKM) method. The *p*-value was used in multiple tests to determine the level of differences in gene expression, with a false discovery rate (FDR) of <0.05. When the FDR < 0.05 and the gene expression exceeded a twofold change (FC), the genes were considered differentially expressed. The high-quality reads were aligned to the genome sequence of *C. crenatum* SYPA 5-5 using Bowtie 2 (http://bowtie-bio.sourceforge.net/bowtie2/index.shtml). The sequences were respectively compared against Clusters of Orthologous Groups (COG), Kyoto Encyclopedia of Genes and Genomes (KEGG), and Gene Ontology (GO) for functional annotation.

### 2.4. DNA Manipulation and Strain Construction

The genomic DNAs were extracted using a bacterial genomic DNA purification kit (Solarbio, Beijing, China). The products of polymerase chain reaction (PCR) were extracted using a DNA gel/PCR purification miniprep kit (Biomiga, Hangzhou, China). The plasmids were purified using a plasmid kit (Omega, Norcross, GA, USA). The primers of this study are listed in Table S2. The genes were deleted through pK18mob*sacB*. The upstream and downstream regions were amplified using PCR extension with PrimeSTAR^®^ Max DNA Polymerase (Takara, Beijing, China), with the PCR steps being 35 cycles of 10 s at 98 °C, 10 s at 55 °C, and 10 s at 72 °C. The upstream and downstream regions of each gene were integrated by overlapping PCR, with the PCR steps being 35 cycles of 10 s at 98 °C, 10 s at 60 °C, and 10 s at 72 °C. The fragment was ligated into pK18mob*sacB* in accordance with the instructions on the NovoRec Plus PCR kit (Novoprotein, Shanghai, China). The vectors were electrically transformed into *C. crenatum*. Frozen intact and osmotically sensitive cells were thawed, spun down for 10 min with 5000 rpm at 4 °C, washed three times with electroporation buffer (10% glycerin), resuspended carefully, and kept on ice. Cells were mixed with plasmid and filled into the electroporation chamber. After the application of two successive pulses, the cells were diluted in a brain–heart infusion medium. The positively engineered mutants were selected from the double crossover clones by using PCR. The three open reading frames of copper-binding peptide (locus_tags are GY20_RS0110270, GY20_RS0100790, and GY20_RS0110545) were amplified using PCR extension with PrimeSTAR^®^ Max DNA Polymerase (Takara, Beijing, China), with the PCR steps being 35 cycles of 10 s at 98 °C, 10 s at 55 °C, and 10 s at 72 °C. The three ORFs were integrated with poly-γ-glutamic acid synthetase A (*pgsA*) from *Bacillus subtilis* by overlapping PCR, with the PCR steps being 35 cycles of 10 s at 98 °C, 10 s at 60 °C, and 10 s at 72 °C. Those fragments were ligated into vector pXMJ19. The recombinant plasmids were electrically transformed into *C. crenatum*.

### 2.5. Quantitative Real-Time PCR (qRT-PCR)

RNA from each sample was diluted to a concentration of 1 mg/mL, and 1 mg of this dilution was used to synthesize cDNA using the PrimeScript^®^RTreagent Kit with gDNA Eraser (TaKaRa, Tokyo, Japan), according to the manufacturer’s protocol. Briefly, RNA was first incubated with 1 mL gDNA Eraser, 2 mL gDNA Eraser Buffer, and RNase-free dH_2_O up to 10 mL at 42 °C for 2 min to remove contaminated cDNA. Subsequently, 1 mL RT Primer Mix, 4 mL PrimeScript^®^ Buffer, 2.1 mL PrimeScript^®^ RT Enzyme Mix I, and RNase-free dH_2_O were added, and the total volume of 20 mL was incubated at 37 °C for 15 min, incubated at 85 °C for 5 s, and finally, cooled on ice. The expression level of the selected genes was validated using qRT-PCR. The 16S rRNA gene of *C. crenatum* was selected as an internal reference. qRT-PCR reactions were carried out using the StepOne Plus Real-Time PCR System (ABI, Carlsbad, CA, USA). The specific primers in qRT-PCR are listed in Table S1. The amplification conditions were as follows: 30 s at 95 °C; 40 cycles of 5 s at 95 °C; 20 s at 60 °C; and 20 s at 72 °C. The data from the qRT-PCR were analyzed according to the 2^−ΔΔCt^ method [14].

### 2.6. Cell Growth Tests

For solid growth tests, the strains were cultured with LB overnight. Then, the media was inoculated into fresh LB. When OD_600nm_ = 1, the strains were diluted by 10^−2^-, 10^−3^-, 10^−4^-, 10^−5^-, and 10^−6^-fold with phosphate-buffered saline. Finally, 5 μL dilutions were spot-plated upon LB solid media. For liquid growth tests, the cells were grown in LB overnight and subsequently diluted in fresh media at a final OD_600nm_. The culture was collected once an hour for OD_600nm_ measurement [15].

### 2.7. Copper Adsorption

The recombinant strains were cultured overnight. Then, the cultures were diluted in a fresh medium and incubated until OD_600nm_ reached about 0.5. The strains were then cultured for 8 h with IPTG. They were collected and washed two times with PBS. The copper adsorption ability was determined using PBS with CuSO_4_ (0.1, 0.5, and 1 mM) for 30 min. The Cu(II) concentrations were measured using an atomic absorption spectrophotometer, an analytical wavelength of 324.8 nm, a lamp current of 2.0 mA, a spectral width of 1.0 nm, and a gas flow of 2000 mL·min^−1^.

### 2.8. ATP and NADP/NADPH Analysis

The culture was collected and immediately centrifuged for 10,000 rmp for 1 min at 4 °C. The collected cells were washed with TE buffer to determine the intracellular ATP concentration. The ATP content was tested using an ATP assay kit (Solarbio, Beijing, China) following the manufacturer’s protocols. The collected cells were washed once with ice-cold PBS to determine the intracellular NADP^+^/NADPH concentration and then 0.5 mL of NADP extraction buffer for NADP^+^ determination or 0.5 mL of NADPH extraction buffer for NADPH determination. Heat extracts were incubated at 80 °C for 5 min, 10,000 rmp was centrifuged at 4 °C for 10 min, and 200 µL of the supernatant was measured using an NADP^+^/NADPH content assay kit (Solarbio, Beijing, China) according to the manufacturer’s protocols.

### 2.9. Bioinformatics and Statistical Analysis

All data were analyzed using one-way ANOVA post hoc comparisons (Tukey’s test, *p* < 0.05, n = 3). Z-score was used to standardize the data and make a correlation curve for qRT–PCR verification and RNA-Seq results. GraphPad Prism 5 was used for all statistical data analysis. All experiments were performed in triplicate, and data are presented as mean ± standard deviation. Three-dimensional structure models were predicted through SWISS-MODEL [16,17].

## 3. Results

### 3.1. DEGs and Enrichment Analysis

**Figure 1:**
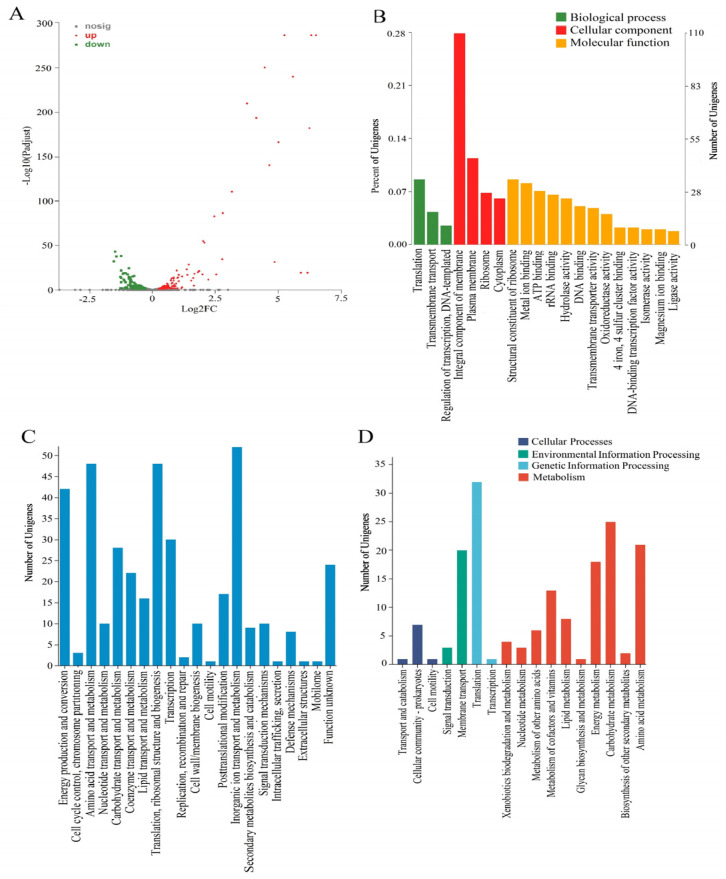
Enrichment analysis of DEGs. (**A**) volcano plot displaying the DEGs, with a false discovery rate (FDR) of <0.05 and |fold change| > 1.2. Green points represent significantly downregulated genes, grey points represent no difference in genes, and red points represent significantly upregulated genes. (**B**) GO classification statistics. (**C**) COG classification statistics. (**D**) top 17 pathways with the largest number of DEGs.

A total of 398 DEGs were identified in the copper-treated group compared with the control (Figure 1A). These DEGs were analyzed using the GO database, which offers three aspects of functional annotation, including biological processes, molecular functions, and cellular components (Figure 1B). In the category of biological process, the DEGs were mainly distributed in “translation”, “transmembrane transport”, and “regulation of transcription, DNA-templated”. In the category of cellular component, the most abundant DEGs fell into the “integral component of membrane” category. In the molecular function category, a high percentage of the DEGs was assigned to “structural constituent of ribosome” and “metal ion binding”. The DEGs were functionally classified into 21 protein families through COG analysis (Figure 1C). The predicted cluster for “inorganic ion transport and metabolism” was the largest group, followed by “translation, ribosomal structure and biogenesis”, “amino acid transport and metabolism”, and “energy production and conversion.” The KEGG database was used to understand the molecular interaction, reaction, and relation networks. The DEGs were mapped to pathways in KEGG for biological function identification. The top 17 pathway classifications are shown in Figure 1D. Among these, “translation” was the most abundant group, followed by “carbohydrate metabolism”, “amino acid metabolism”, “membrane transport”, and “energy metabolism.”

### 3.2. Effects of Copper on Energy Metabolism

**Figure 2:**
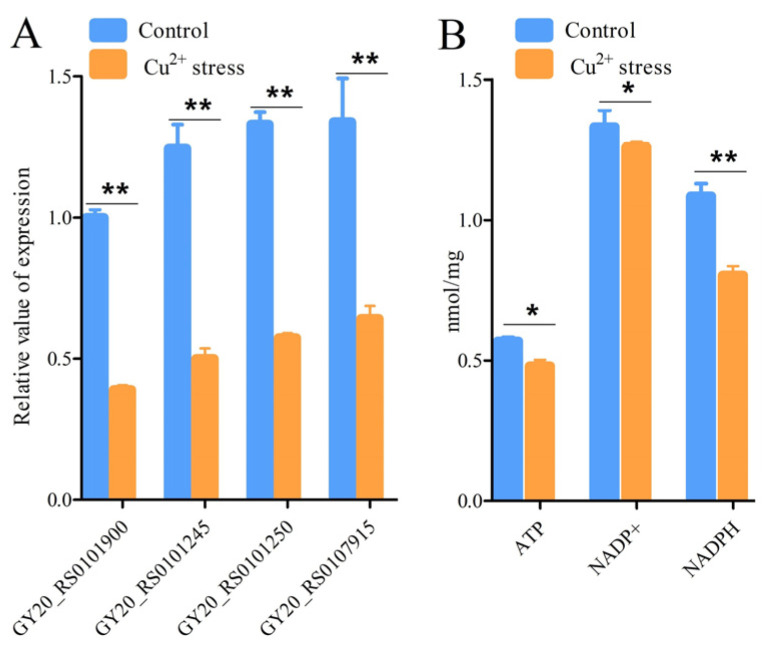
Effects of copper on energy metabolism. (**A**) qRT-PCR of DEGs related to energy metabolism. GY20_RS0101900, GY20_RS0101245, GY20_RS0101250, and GY20_RS0107915 encode succinate dehydrogenase/fumarate reductase iron-sulfur subunit, succinate dehydrogenase cytochrome b subunit, succinate dehydrogenase (quinone) flavoprotein subunit, and aconitate hydratase, respectively. (**B**) Concentrations of ATP, NADP+, and NADPH. “*” indicates *p*-value of <0.05, “**” indicates *p*-value of <0.001.

The results of the enrichment analysis showed significant changes in the expression of energy metabolism-related genes in the copper stress group versus the control group. Four significantly downregulated DEGs related to energy metabolism, which included three succinate dehydrogenase genes (GY20_RS0101900, GY20_RS0101245, GY20_RS0101250) and one aconitate hydratase gene (GY20_RS0107915), were selected to conduct the qRT-PCR. The log2 FC of these DEGs ranged between −1.2 and −1.5 (Table S3). The qRT-PCR results showed that the expression levels of the selected four genes were suppressed by copper stress (Figure 2A). These results further confirmed the reproducibility and reliability of transcriptome analysis. In addition, the concentrations of ATP, NADP^+^, and NADPH were measured (Figure 2B). The copper stress group exhibited low ATP, NADP^+^, and NADPH, and the NADPH decreased by 35% compared with that of the control group.

### 3.3. Effects of Copper on Metal Ion Transport

**Figure 3:**
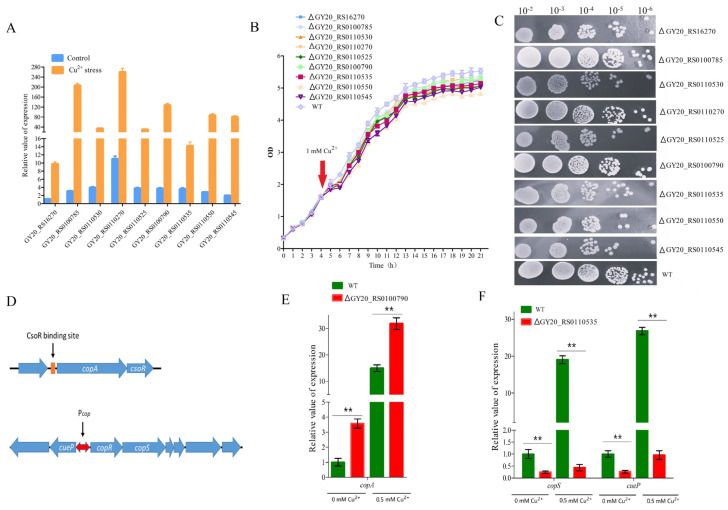
Effects of copper on metal ion transport. (**A**) qRT−PCR of DEGs related to metal ion transport. (**B**) Liquid growth analysis. (**C**) Solid growth analysis. (**D**) Gene arrangements of *copS* -and *copR*−related operons. (**E**) Relative transcription level of *copA* in *cosR* deletion mutant and wild type. (**F**) Relative transcription level of *copS* and *cueP* in *copR* deletion mutant and wild type.

The enrichment analysis results showed significant changes in the expression of metal ion transport-related genes in the copper stress group versus the control group. A total of nine significantly upregulated DEGs related to metal ion transport were selected to conduct qRT-PCR. The log2 FC of these DEGs ranged between 2.5 and 6.5 (Table S4). The results of the qRT-PCR showed that the transcription of these genes increased greatly under copper stress (Figure 3A). Particularly, the expression of GY20_RS0100785 increased by nearly 70-fold compared with that of the control. Then, whether the growth of the nine DEG-deleted phenotypes differed from that of the wild-type in response to copper stress was explored. The wild-type strain and the deleted phenotypes were cultured for growth analyses. The growth of all DEG-deleted phenotypes was slightly weaker than that of the wild-type in a liquid medium. Among these, the GY20_RS0110550-deleted phenotype exhibited the lowest growth yields at the final stage (Figure 3B). Compared with those in the wild type, the growth of the GY20_RS0100785-, GY20_RS0110270-, and GY20_RS0100790-deleted phenotypes slightly weakened; the colonies of the GY20_RS0110550- and GY20_RS0110545-deleted phenotypes showed a marked decrease; and the colonies of the GY20_RS0110530-deleted phenotype decreased (Figure 3C). These results indicated that the nine DEGs were required for *C. crenatum* growth under copper stress.

Among the nine DEGs related to metal ion transport, GY20_RS0100790 and GY20_RS0110535 are transcriptional regulators. The multiple homologous sequence alignment indicated that GY20_RS0100790 is a homolog of *csoR* and GY20_RS0110535 is a homolog of *copR*. In *Corynebacterium glutamicum*, *csoR* and *copR* were found to play important roles in copper resistance. CopR binds to the DNA region, resulting in the transcriptional activation of upstream and downstream operons containing genes that encode copper resistance proteins. CsoR binds to the DNA region and derepresses the transcription of downstream operons, which is part of the copper detoxification process (Figure 3D). The *cueP* and *copS* expression levels of ΔGY20_RS0110535 and the *copA* expression of the ΔGY20_RS0110790 were compared to those of the wild type by using qRT-PCR to identify the regulon of the response regulators GY20_RS0110535 and GY20_RS0110790 in *C. crenatum*. The mRNA level of *copA* nearly increased by two- to threefold with or without copper due to GY20_RS0110790 deletion. The *copA* expression in the wild strain cultured with copper increased by 15-fold compared with that cultured without copper. However, the *copA* expression in the ΔGY20_RS0110790 cultured with copper by only ninefold compared with that cultured without copper (Figure 3E).

The mRNA level of *copS* and *cueP* nearly decreased by 78%–98% due to GY20_RS0110535 deletion. The *copS* and *cueP* expression levels in ΔGY20_RS0110535 cultured with copper decreased by only two- to threefold compared with that cultured without copper. However, the *copS* and *cueP* expression levels in the wild type cultured with copper decreased by 19- to 26-fold compared with that cultured without copper (Figure 3F). Therefore, the GY20_RS0110790 and GY20_RS0110535 of *C. crenatum* functioned as a copper sensor and regulated the gene expression.

### 3.4. Cell-Surface Display of Copper-Binding Peptides Influencing Growth Rate in Copper Stress

**Figure 4:**
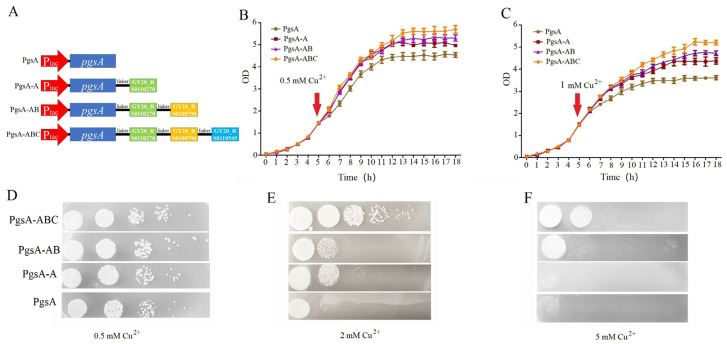
Cell-surface display of copper-binding peptides influencing growth rate in copper stress. (**A**) Schematic of cell-surface display of copper-binding peptides. The linker region was “GSG”. (**B**,**C**) Liquid growth analysis. Strains were cultured in LB for 4 h and subsequently added into 0.5 mM or 1 mM copper ions. Then, 3 mL of media was collected every 1 h to measure OD. (**D**–**F**) Solid growth analysis.

According to protein sequence and structure analysis, GY20_RS0110270, GY20_RS0100790, and GY20_RS0110545 encoding proteins consist of 67, 100, and 78 amino acids, respectively, and all contain a copper-binding domain. Cell-surface modification results in specificity binding with metals owing to the proteins containing metal-binding motifs fusing with anchoring proteins on the cell surface [18]. PgsA is a constituent protein of the polyglutamic acid synthetase system of *B. subtilis*, which can firmly anchor certain enzymatic systems to cell walls, and different proteins were displayed successfully on the surface of the cell using PgsA as an anchor motif [19]. The three proteins were ligated individually and then anchored to the *C. crenatum* surface by using PgsA as a passenger protein (Figure 4A). Then, whether the growth of the surfaced-displayed strains differs from that of the control strain during copper stress was evaluated. The results of liquid growth analyses showed obvious differences in growth (Figure 4B,C), and the cell surface-displayed strains showed higher growth rates, indicating that the cell-surface display of copper-binding peptides contributes to copper stress tolerance. In addition, the cell surface-displayed strains showed similar increases in growth observed in solid media with different copper concentrations (Figure 4D–F). The number of colonies in the control was less than that of the cell surface-displayed strains with copper stress. When 5 mM of copper was added, the groups of PgsA and PgsA-A barely formed visible colonies. The group of PgsA-ABC exhibited the highest growth rates in liquid and solid media with copper stress. This result suggested that the displayed peptides can interact with environmental copper, resulting in the formation of barriers to prevent copper from being transported into the cell.

### 3.5. Copper Adsorption

**Figure 5:**
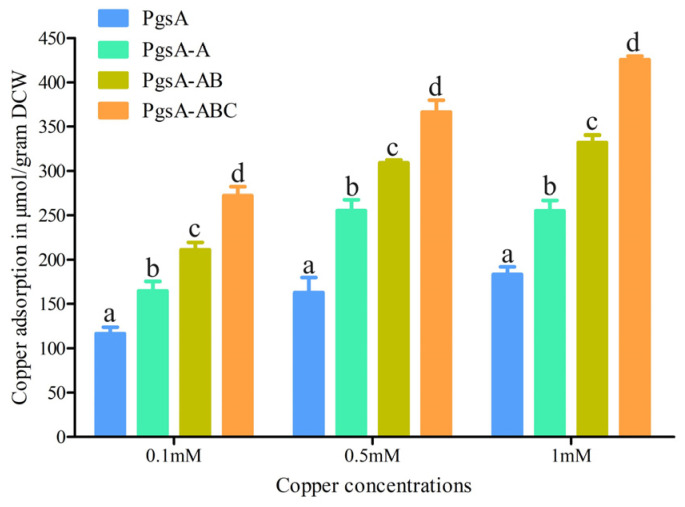
Evaluation of copper adsorption. The calculated data (mean ± SD) of three individuals (n = 3) with different letters (a, b, c, d) were significantly different (*p* < 0.05) between different strains at the same copper concentration.

The surface-displayed strains and the control were incubated with 0.1, 0.5, and 1 mM Cu^2+^ for 30 min separately to investigate the copper adsorption capacity of the surface display system. In general, when the copper concentration was increased from 0.1 mM to 1 mM, the amount of adsorbed copper increased, and more copper was adsorbed by the cell surface-displayed strains than the control strains (Figure 5). The maximum copper adsorption of 425.62 μmol/g dry cell weight (DCW) was achieved by PgsA-ABC with a copper concentration of 1 mM. These results demonstrated that the copper adsorption capacity was enhanced by the display of multiple copper-binding peptides.

## 4. Discussion

The survival of strains under heavy metal stress is important for heavy metal remediation. *E. coli*, *P. aeruginosa*, *Bacillus cereus*, and *B. subtilis* showed apparent reductions in growth rates with 0.1 mM copper [20]. *E. coli* K-12 MG1655 demonstrated growth retardation with 0.78 mM copper [21]. *Corynebacterium glutamicum* is a close relative of *C. crenatum*, and its growth rate tested with 0.5 mM copper decreased by 40% compared with that tested without copper [22]. *Halomonas* spp. strain MA2, a Gram-negative halophilic bacterium, was resistant to a wide range of toxic metals, the growth of strain MA2 with 1 mM copper was obviously slower [23]. *Cupriavidus metallidurans*, a type of strain for studying metal resistance, was isolated in metal-contaminated industrial environments. Its growth rate, tested with over 0.75 mM copper, decreased by 60% compared with that tested without copper [24]. However, the growth of *C. crenatum* with 0.5 and 1 mM copper was slightly slower than that of *C. crenatum* without copper (Figure S1). This result suggested that *C. crenatum* possesses a better tolerance in resistance to copper than the other strains. However, more than 50% of the isolated bacteria from fresh produce had minimal inhibitory concentrations (MICs) for copper greater than 16 mM [25]. The copper tolerance of *C. crenatum* was still slower than that of copper-resistant strains screened from the wild.

The transcriptomic responses to copper stress were investigated to analyze the copper resistance mechanisms in *C. crenatum*. Living cells can remove metal ions through transmembrane transport [26]. Many DEGs were found to be classified as “transmembrane transport” in the category of biological process. The transcriptome in *Cupriavidus gilardii* CR3 exposed to copper also showed that 18 genes were related to membrane transport were DEGs [6]. Furthermore, the most abundant DEGs fell into the “integral component of membrane” in the category of cellular component. The results indicated that the membrane component plays an important role in the copper resistance of *C. crenatum*. Meanwhile, bacteria are known for diverse heavy metal resistance systems, including *cus*, *cue*, *cop*, and *poc* systems [6]. In this work, the expression levels of *cus-*, *cop-*, and *cue*-related genes between the copper stress group and the control were obviously upregulated (Table S4). The qRT-PCR results showed that the transcription of these genes increased greatly in response to copper stress, and the growth of the gene-deleted phenotypes was weaker than that of the wild type. Thus, the *cus*, *cop*, and *cue* systems were considered important copper resistance mechanisms in *C. crenatum*. Extracellular polymeric substances (EPS) play a crucial role in heavy metal bio-adsorption using activated sludge, but transcriptomic analysis makes it difficult to directly explore the interaction mechanism between heavy metals and EPS.

In addition, marked downregulation was found in some energy metabolism-related genes in the copper stress group versus the control group, including three succinate dehydrogenase genes and one aconitate hydratase gene (Table S3). These results are very similar to *Candida albicans* [27]. The tricarboxylic acid cycle (TAC) is the central pathway of energy transfer for organisms. It generates NADH and FADH_2_ for ATP production by the electron transport chain [28]. The catalyzed reaction of aconitate hydratase is a reversible isomerization of citrate into isocitrate, which is one of the initial stages of TAC. Succinate dehydrogenase catalyzes succinate oxidation in TAC and transfers the electrons to the electron transport chain. The copper stress group exhibited low ATP and NADP^+^; moreover, the NADPH decreased by 35% compared with that of the control. These results showed that energy metabolism disturbance may be one of the reasons why *C. crenatum* showed low growth under copper stress.

Heavy metal response genes are crucial for microbial utilization in heavy metal remediation. Here, two copper response transcriptional regulators and three copper-binding peptides in *C. crenatum* were investigated. The CsoR and CopR of *C. crenatum* can be used as a copper sensor in microbiological copper treatments. The three copper-binding peptides were ligated individually and then anchored on the surface of *C. crenatum* by using PgsA as a passenger protein. The displayed peptides can interact with environmental copper, resulting in the formation of barriers to prevent copper from being transported into the cell. The cell surface-displayed strains demonstrated higher growth rates, and their maximum copper adsorption was 425.62 μmol/g DCW.

## 5. Conclusions

The *cus*, *cop*, and *cue* systems were considered important copper resistance mechanisms in *C. crenatum*. Energy metabolism disturbance may be one of the reasons why *C. crenatum* showed low growth under copper stress. The copper response genes of *C. crenatum* could be useful for the construction of a copper response gene circuit for the enhanced bioremediation and detection of copper. Overall, this investigation provided the basis for utilizing *C. crenatum* for copper pollution control.

## Data Availability

Data are contained within the article and Supplementary Materials.

## Supplementary Materials

The following supporting information can be downloaded at https://www.mdpi.com/article/10.3390/microorganisms12050951/s1: Figure S1: Growth of *C. crenatum* under different concentrations of copper; Table S1: Strains and plasmids in this study; Table S2: Primers and sequences in this study; Table S3: Selected DEGs’ related energy metabolisms; Table S4: Selected DEGs’ related metal ion transports.
