# Supplementary material for: Copper Resistance Mechanism and Copper Response Genes in Corynebacterium crenatum
Source: Microorganisms. 2024 May 8;12(5):951. doi: 10.3390/microorganisms12050951 (PMC11124244; doi:10.3390/microorganisms12050951)
Supplement: Supplementary file 1 [file microorganisms-12-00951-s001.zip › microorganisms-2963699-supplementary.pdf]

Table S1. Strains and plasmids in this study.

Strains or plasmids	characteristics/purposes	Source
Plasmids		
pK18mob*sacB*	Mobilizable vector, allows for selection of double crossover in *C. crenatum*, Km^R, *sacB*	Biovector
pXMJ19	Shuttle vector for overexpression, Chl^R	Biovector
pK18mob*sacB*-Δ6270	A derivative of pK18mob*sacB*, harboring the fragment of GY20_RS16270 deletion	This work
pK18mob*sacB*-Δ785	A derivative of pK18mob*sacB*, harboring the fragment of GY20_RS0100785 deletion	This work
pK18mob*sacB*-Δ530	A derivative of pK18mob*sacB*, harboring the fragment of GY20_RS0110530 deletion	This work
pK18mob*sacB*-Δ270	A derivative of pK18mob*sacB*, harboring the fragment of GY20_RS0110270 deletion	This work
pK18mob*sacB*-Δ525	A derivative of pK18mob*sacB*, harboring the fragment of GY20_RS0110525 deletion	This work
pK18mob*sacB*-Δ790	A derivative of pK18mob*sacB*, harboring the fragment of GY20_RS0100790 deletion	This work
pK18mob*sacB*-Δ535	A derivative of pK18mob*sacB*, harboring the fragment of GY20_RS0110535 deletion	This work
pK18mob*sacB*-Δ550	A derivative of pK18mob*sacB*, harboring the fragment of GY20_RS0110550 deletion	This work
pK18mob*sacB*-Δ545	A derivative of pK18mob*sacB*, harboring the fragment of GY20_RS0110545 deletion	This work
pXMJ19-*pgsA*	A derivative of pXMJ19, harboring overlap fragment of *pgsA*	This work
pXMJ19-*pgsA*-A	A derivative of pXMJ19, harboring overlap fragment of *pgsA* and GY20_RS0110270	This work
pXMJ19-*pgsA*-AB	A derivative of pXMJ19, harboring overlap fragment of *pgsA,* GY20_RS0110270 and GY20_RS0100790	This work
pXMJ19-*pgsA*-ABC	A derivative of pXMJ19, harboring overlap fragment of *pgsA*, GY20_RS0110270, GY20_RS0100790 and GY20_RS0110545	This work
Strains		
E. coli DH5α	Clone host strain	Invitrogen
WT	*C. crenatum* AS 1.542 wild-type strain	China center of industrial culture collection
ΔGY20_RS16270	*C. crenatum* AS 1.542 with a deletion of GY20_RS16270	This work

ΔGY20_RS0100785	*C. crenatum* AS 1.542 with a deletion of GY20_RS0100785	This work
ΔGY20_RS0110530	*C. crenatum* AS 1.542 with a deletion of GY20_RS0110530	This work
ΔGY20_RS0110270	*C. crenatum* AS 1.542 with a deletion of GY20_RS0110270	This work
ΔGY20_RS0110525	*C. crenatum* AS 1.542 with a deletion of GY20_RS0110525	This work
ΔGY20_RS0100790	*C. crenatum* AS 1.542 with a deletion of GY20_RS0100790	This work
ΔGY20_RS0110535	*C. crenatum* AS 1.542 with a deletion of GY20_RS0110535	This work
ΔGY20_RS0110550	*C. crenatum* AS 1.542 with a deletion of GY20_RS0110550	This work
ΔGY20_RS0110545	*C. crenatum* AS 1.542 with a deletion of GY20_RS0110545	This work
PgsA	*C. crenatum* AS 1.542 harboring plasmids of pXMJ19-*pgsA*	
PgsA-A	*C. crenatum* AS 1.542 harboring plasmids of pXMJ19-*pgsA*-A	This work
PgsA-AB	*C. crenatum* AS 1.542 harboring plasmids of pXMJ19-*pgsA*-AB	This work
PgsA-ABC	*C. crenatum* AS 1.542 harboring plasmids of pXMJ19-*pgsA*-ABC	This work

Table S2. Primers and sequences in this study.

primers	Primer sequences （5′-3′）	Target
6270-up-F	gattacgaattcgagctcggtacgaagcagtactttagg	Upstream fragment of GY20_RS16270
6270 -up-R	gtgttctccatgcttatgaaagtactttcctgtagtaagtgaatgag	Upstream fragment of GY20_RS16270
6270-down-F	ctcattcacttactacaggaaagtactttcataagcatggagaacac	Downstream fragment of GY20_RS16270
6270-down-R	ttttcccagtcacgacgttgttatgcgcgcttctccatc	Downstream fragment of GY20_RS16270
785-up-F	gattacgaattcgagctcggtatggctcagacacccgc	Upstream fragment of GY20_RS0100785
785-up-R	ctcatccatcgtggaggccagtacggcccactgcc	Upstream fragment of GY20_RS0100785
785-down-F	ggcagtgggccgtactggcctccacgatggatgag	Downstream fragment of GY20_RS0100785
785-down-R	ttttcccagtcacgacgttgtactgtgcgtccgtgacca	Downstream fragment of GY20_RS0100785
530-up-F	gattacgaattcgagctcggttgaggtaggaggccaccatct	Upstream fragment of GY20_RS0110530
530-up-R	aggccatgggtagtcaggaatgtcaatctccttggatcgtggtaag	Upstream fragment of GY20_RS0110530
530-down-F	cttaccacgatccaaggagattgacattcctgactacccatggcct	Downstream fragment of GY20_RS0110530
530-down-R	ttttcccagtcacgacgttgttgctgaggcatcaggtcagg	Downstream fragment of GY20_RS0110530
270-up-F	gattacgaattcgagctcggtcaccaatgccataaacatctaaatgc tg	Upstream fragment of GY20_RS0110270
270-up-R	ggcaccggtttctagggtgattattggcccctttctttcaggt	Upstream fragment of GY20_RS0110270
270-down-F	acctgaaagaaaggggccaataatcaccctagaaaccggtgcc	Downstream fragment of GY20_RS0110270
270-down-R	ttttcccagtcacgacgttgtggcgcccgagcagat	Downstream fragment of GY20_RS0110270
525-up-F	gattacgaattcgagctcggtatgacaagtagtttttcccggc	Upstream fragment of GY20_RS0110525
525-up-R	tggtccactcgacgtcctgcaggttcaatggggtcctggg	Upstream fragment of GY20_RS0110525
525-down-F	cccaggaccccattgaacctgcaggacgtcgagtggacca	Downstream fragment of GY20_RS0110525

525-down-R	ttttcccagtcacgacgttgtccatcgacaggtagatcgattcgg	Downstream fragment of GY20_RS0110525
790-up-F	gattacgaattcgagctcggtaaggaactgggactcacccc	Upstream fragment of GY20_RS0100790
790-up-R	cggagatctgcgtgaggatgtagttgctccttgctttgaatcctcg	Upstream fragment of GY20_RS0100790
790-down-F	cgaggattcaaagcaaggagcaactacatcctcacgcagatctccg	Downstream fragment of GY20_RS0100790
790-down-R	ttttcccagtcacgacgttgttccattttgagggtacggattcgg	Downstream fragment of GY20_RS0100790
535-up-F	gattacgaattcgagctcggtaccccgtgagggcgag	Upstream fragment of GY20_RS0110535
535-up-R	agatccagcaccacaacatcgagaccccagtatcgcttcg	Upstream fragment of GY20_RS0110535
535-down-F	cgaagcgatactggggtctcgatgttgtggtgctggatct	Downstream fragment of GY20_RS0110535
535-down-R	ttttcccagtcacgacgttgtacatcgacgatgcgttcatcg	Downstream fragment of GY20_RS0110535
550-up-F	gattacgaattcgagctcggtatgagcactccccaccacc	Upstream fragment of GY20_RS0110550
550-up-R	cgacgaccttctcggcccgccgtagaggaagatgatcg	Upstream fragment of GY20_RS0110550
550-down-F	cgatcatcttcctctacggcgggccgagaaggtcgtcg	Downstream fragment of GY20_RS0110550
550-down-R	ttttcccagtcacgacgttgtcgatcagggcgaaccagaaca	Downstream fragment of GY20_RS0110550
545-up-F	gattacgaattcgagctcggtacagggggcgtccac	Upstream fragment of GY20_RS0110545
545-up-R	gtcggggtcgagatgatgggggccggggcggaga	Upstream fragment of GY20_RS0110545
545-down-F	tctccgccccggcccccatcatctcgaccccgac	Downstream fragment of GY20_RS0110545
545-down-R	ttttcccagtcacgacgttgtgataaacgccacggtgatcgc	Downstream fragment of GY20_RS0110545
pgsA-F	attaattaagcttgcatgcctatgaaaaaagaactgagctttcat	ORF of *pgsA*
pgsA-R	ctgaattcgagctcggtacccttatttagattttagtttgtcact	ORF of *pgsA*
790-F	agtgacaaactaaaatctaaaggcagcggtatgtccaacagcgaatgcca	ORF of GY20_RS0100790
790-R	ctgaattcgagctcggtacccttaggccttactgaagcg	ORF of GY20_RS0100790

545-F	cgcttcagtaaggccggcagcggtgtggccgcggcaaccgacgc a	ORF of GY20_RS0110545
545-R	ctgaattcgagctcggtacccttaggacaagacggtgtagccggt	ORF of GY20_RS0110545
270-F	accggctacaccgtcttgtccggcagcggtatggctatcaagaact acactg	ORF of GY20_RS0110270
270-R	ctgaattcgagctcggtacccttatgcaacaaccttgtagccagcc	ORF of GY20_RS0110270
6270-Q-F	gcatgatgctgttgattgcg	To detect the gene expression levels of
6270-Q-R	ttctagccaatgtcccagca	To detect the gene expression levels of
785-Q-F	caagggctccaaagttaccg	To detect the gene expression levels of
785-Q-R	cgatgacaacgggaacgaaa	o detect the gene expression levels of
530-Q-F	cttctaccacagcctcacga	To detect the gene expression levels of
530-Q-R	tgctcatcgatcaggacctc	To detect the gene expression levels of
270-Q-F	tggacgtcaccctagaaacc	To detect the gene expression levels of
270-Q-R	aacaaccttgtagccagcct	To detect the gene expression levels of
525-Q-F	ccaatgaactgcctgagagc	To detect the gene expression levels of
525-Q-R	ccggtgtgggaatggtagaa	To detect the gene expression levels of
790-Q-F	atcgacatcctcacgcagat	To detect the gene expression levels of
790-Q-R	cgaggtcagctgcttctttg	To detect the gene expression levels of
535-Q-F	caccttctcggactgctaca	To detect the gene expression levels of
535-Q-R	accagttcccggatgctaaa	To detect the gene expression levels of
550-Q-F	gtggtcctagccagtgatga	To detect the gene expression levels of
550-Q-R	cagaccaggttctggaccat	To detect the gene expression levels of
545-Q-F	gtctccatcgtcacggtca	To detect the gene expression levels of

545-Q-R	gtgtagccggtctcctcaat	To detect the gene expression levels of
16S-Q-F	ctgctgcaagaccatccttc	To detect the gene expression levels of
16S-Q-R	tggtgacgagggcaagtatt	To detect the gene expression levels of
pXMJ19 check-F	cggctcgtataatgtgtgga	Recombinant pXMJ19 vector detecting
pXMJ19 check-R	atcttctctcatccgccaaa	Recombinant pXMJ19 vector detecting
M13-F	cgccagggttttcccagtcacgac	Recombinant pK18*mobsacB* vector [illegible]
M13-R	gaggggataacaatttcacacagg	Recombinant pK18*mobsacB* vector

Table S3. The selected DEGs related energy metabolism.

Gene_id	Gene description	Log2FC
GY20_RS0101900	succinate dehydrogenase iron-sulfur subunit, *sdhB*	-1.536938671
GY20_RS0101245	succinate dehydrogenase cytochrome b subunit, *sdhC*	-1.493861784
GY20_RS0101250	succinate dehydrogenase (quinone) flavoprotein subunit, *sdhA*	-1.43069148
GY20_RS0107915	aconitate hydratase, *acnA*	-1.249445956

Table S4. The selected DEGs related metal ion transport.

Gene_id	Gene description	Log2FC
GY20_RS0110535	response regulator transcription factor, the homologue of *copR*	2.450084028
GY20_RS0110270	cation transporter, the homologue of *copZ*	3.759152948
GY20_RS0110525	multicopper oxidase family protein, the homologue of *copO*	4.124230665
GY20_RS0110530	metal-binding protein, the homologue of *cueP*	4.466062036
GY20_RS0100790	metal-sensitive transcriptional regulator, the homologue of *cosR*	5.009155814
GY20_RS16270	heavy metal translocating P-type ATPase, the homologue of *copB*	5.231444696
GY20_RS0110550	copper-translocating P-type ATPase, the homologue of *copB*	5.578069165
GY20_RS0110545	heavy-metal-associated domain-containing protein, the homologue of *copZ*	6.224351036
GY20_RS0100785	heavy metal translocating P-type ATPase, the homologue of *copA*	6.484716556

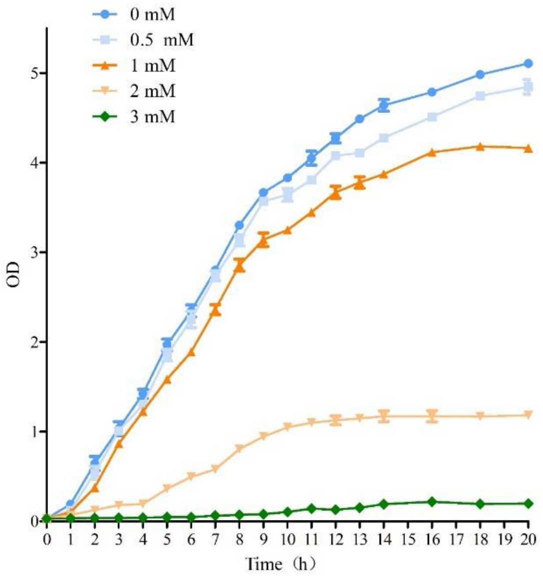

Figure S1. Growth of *C. crenatum* under different concentrations of copper.

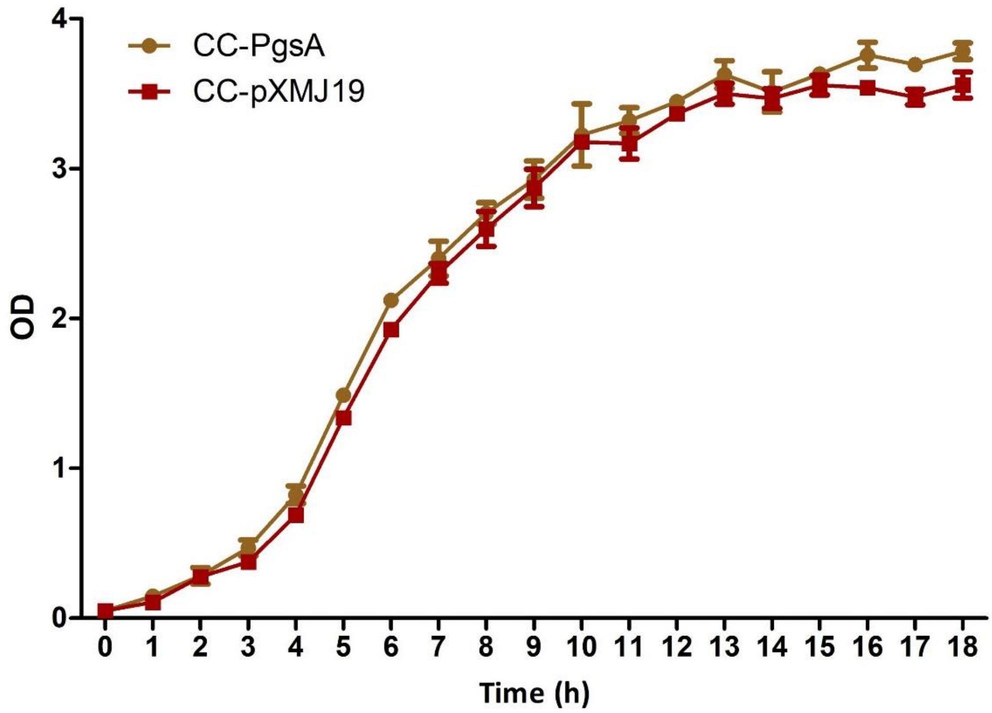

Figure S2. Growth of *the strain* harboring empty vector (CC-pXMJ19) and the strain expressing pgsA (CC-PgsA) under 1 mM copper.
